# A Novel Methodology to Obtain the Mechanical Properties of Membranes by Means of Dynamic Tests

**DOI:** 10.3390/membranes12030288

**Published:** 2022-03-02

**Authors:** Antonia Lima-Rodriguez, Jose Garcia-Manrique, Wei Dong, Antonio Gonzalez-Herrera

**Affiliations:** 1 Department of Civil Engineering, Materials and Manufacturing, School of Engineering, University of Malaga, C/Arquitecto Francisco Peñalosa 6, 29071 Malaga, Spain; josegmo@uma.es (J.G.-M.); agh@uma.es (A.G.-H.); 2 VA Loma Linda Healthcare System and Department of Otolaryngology—Head & Neck Surgery, Loma Linda University Health, 11234 Anderson St., Loma Linda, CA 92354, USA; wei.dong@va.gov

**Keywords:** membrane characterization, experiment, finite element model

## Abstract

A new, non-destructive methodology is proposed in this work in order to determine the mechanical properties of membrane using vibro-acoustic tests. This procedure is based on the dynamic analysis of the behavior of the membrane. When the membrane is subjected to a sound excitation it responds by vibrating based on its modal characteristics and this modal parameter is directly related to its mechanical properties. The paper is structured in two parts. First, the theoretical bases of the test are presented. The interaction between the sound waves and the membrane (mechano-acoustic coupling) is complex and requires meticulous study. It was broadly studied by means of numerical simulations. A summary of this study is shown. Aspects, such as the position of the sound source, the measuring points, the dimensions of the membrane, the frequency range, and the magnitudes to be measured, among others, were evaluated. The validity of modal analysis curve-fitting techniques to extract the modal parameter from the data measures was also explored. In the second part, an experimental test was performed to evaluate the validity of the method. A membrane of the same material with three different diameters was measured with the aim of estimating the value of the Young’s modulus. The procedure was applied and satisfactory results were obtained. Additionally, the experiment shed light on aspects that must be taken account in future experiments.

## 1. Introduction

Thin film materials are elements present in multitude of engineering problems, such as the study of microdevices or biomechanics, and their characterization is essential to predicting and simulating their behavior. Nevertheless, the determination of the mechanical properties of a material is a difficult problem to solve when it is in its membrane form. These materials present a small thickness and low stiffness. Traditional experimental tests present inaccuracies in the characterization of some of these materials and mostly involve the destructive testing of the material.

One of the best-known tests to determine mechanical properties is the tensile test. With this test, the Young’s modulus is determined, among other mechanical characteristics, and it is a destructive test. Many authors have used this test to determine the Young’s modulus of thin film materials of different compositions. In [1], both the tensile test and resonance techniques were used to mechanically characterize boron-doped silicon films with thicknesses of 1 to 4 µm. In [2], the Young’s modulus of fetal preterm and term amniotic membranes were measured by using a stress-strain apparatus. In [3], thin micromembranes were subjected to compressive stresses, with or without initial deflections, to determine the Young’s modulus and the residual stress of the micromembranes for use in microdevices.

Other works are based on the relationship between deflection and pressure. In [4], this relationship was used to obtain the Young’s modulus and internal stress of composite rectangular membranes of 2 mm by 8 mm.

Resonance methods are used in the literature to mechanically characterize thin film materials [5,6,7,8,9]. In [5,6,9], cantilever beams made from thin films were forced to vibrate. Young’s moduli were calculated by measuring the resonance frequencies of the beams. In [7], the influence on Young’s modulus of aging phenomena in heavily doped micromachined silicon cantilever beams was studied. In [8], the first and second symmetric vibration modes of a circular diaphragm thin film were measured to calculate Young’s modulus and the residual stress of the film, based on the resolution of the Rayleigh–Ritz equation, analytically.

Focusing on the field of interest of the authors, the study of the tympanic membrane, there are quite a few studies carried out to determine the Young’s modulus. The function of this membrane is very important in the hearing process, since it is responsible for transmitting the sound waves captured from the external auditory canal into the ear. One of the most important parameters to obtain a physical model of this membrane is the elastic modulus. Authors have usually carried out experiments with samples cut from the tympanic membrane to characterize them mechanically. In [10], the Young’s modulus of a strip of human tympanic membrane was measured using a longitudinal vibration technique, while ref. [11] performed a beam bending test and determined the Young’s modulus and ref. [12] reported results of uniaxial tests of the human eardrum. In [13,14], the tensile test was applied to eardrum specimens from fresh human cadavers. In [13], the Young’s modulus was determined through mechanical tension tests and a t test was used to evaluate it statistically. In [14], the Ogden hyperelastic model and the digital image correlation method were used to analyze the experimental results. Recently, ref. [15] compared the acoustic behavior of different types of membranes for use in the repair of chronic tympanic membrane perforations. Membranes of different thicknesses (10–100 µm) were tested in a model with a simulated ear canal to determine the frequency response function (FRF) and their resistance to pressure loads. The tensile test was used to obtain the mechanical rigidity to pressure loads.

Although these measurement methods can be useful on certain occasions, they also have certain limitations. As the tympanic membrane is a biological material, it is not always possible to have enough samples to carry out a destructive test, and measurements cannot be carried out in situ. Furthermore, another aspect to consider is that the results are subject to significant uncertainties due to the heterogeneous composition of the tympanic membrane and its anisotropic properties. In fact, the range of Young’s modulus results for the tympanic membrane is very broad, even of different orders of magnitude in many cases, due to the alteration of the material during the experiment.

There are interesting works focused on nano- or micro-indentations for the mechanical characterization of tympanic membranes [16,17,18,19,20]. In [19], the material was also subjected to a uniaxial tensile test to validate the results obtained, and in [20], the tissue indentation technique and an inverse finite element analysis were used to estimate the Young’s modulus of the tympanic membrane. Other authors have also used this technique to calculate the elastic modulus for other types of film-type materials [21,22].

The nano- or micro-indentation method presents good results to obtain the strain–stress curve of the membrane. Nonlinear material behavior with a large strain can be captured, but less information is obtained at the low strain range. The problem is that it is precisely at that low strain range where most membranes behave in vibrations due to acosutic waves.

Another technique used to calculate the mechanical properties of materials is AFM force spectroscopy [23,24,25]. In these works, the calculation of Young’s modulus is focused on microscopic materials. In these cases, it may be interesting to monitor the state of a sample during the test, to be sure that unwanted failures do not occur in the material [26,27].

There are other works that study the mechanical behavior of the tympanic membrane using resonance methods [28,29]. In [28], in addition to this dynamic method, used a constitutive model to estimate the properties based on known stiffness. After that, the existing experimental data were reinterpreted using the classical theory of composite lamination.

In [29], a new Hopkinson tension bar was developed to investigate the mechanical behavior of the tympanic membrane at high strain rates.

The ASTM E 756-05 standard [30] presents an experiment that enables the determination dynamic properties, such as natural frequencies and the damping of the material, and then establishes analytical relationships with mechanical properties, such as Young’s modulus (E) or the transverse modulus (G). The tests were based on the analysis of the behavior of an excited bracket by means of a vibration in a frequency range of 50–5000 Hz at different temperatures. In the case of non-self-supporting materials, such as the case described here, it uses a configuration where it is attached to a base material with sufficient rigidity. In these cases, the test is repeated with the composite piece and with the base material, to establish relationships between the damping of the second versus the set. The need to join both materials, the inclusion of adhesives and the simplifications assumed imply precision levels that the standard establishes at 20%. Although they are a starting point, the properties of materials change when they are deployed in film or membrane forms.

Due to the need for a test to determine the mechanical characteristics of thin film materials, a non-destructive methodology is proposed to determine the Young’s modulus using vibro-acoustic tests, based on the dynamic analysis of the behavior of the membrane. The tested sample remains intact and can be reused in other configurations or tests in the future. A membrane of a material subjected to a sound excitation responds by vibrating in a range of small deformations and small displacements, where bending is the main mechanical behavior. This vibration depends on the dynamic characteristics of the membrane in terms of resonance frequencies and vibration modes. The main purpose of the methodology is to relate the expected response to a controlled excitation of a specimen based on its mechanical properties, specifically to obtain the Young’s modulus information.

However, this measurement is not easy to obtain. The interaction between the sound waves and the response of the membrane (mechanical acoustic coupling) is complex and requires a meticulous study. The response is affected by a multitude of factors whose influence must be studied. The position of the sound source, the measuring points, the dimensions of the membrane, the sound frequencies or the magnitudes to be measured, among others, must be considered.

The methodology proposed by the authors is based on a controlled experiment where the range of sound pressure on the membrane guarantees the vibrations exhibit small-strain behavior and present a linear and elastic response. In order to avoid sources of uncertainty as much as possible, the main parameters were studied by previous tests or numerical simulations with Ansys software. Once the experiment procedure was established, different strategies of data post-processing were analyzed, including direct observation and Nyquist diagrams [31]. The experiment configuration (circular specimens) enables the use of a known analytical solution to describe the dynamic response, which has been widely covered in the literature for extreme cases of plate and membrane behaviors [32,33] and for intermediate situations [34,35]. Finally, experimental displacement measurement at strategic point of the specimen identified a group of main frequencies that, introduced in the analytical formulation, determined the mechanical properties.

The paper is structured as follows. In the materials and methods section, the general description of the methodology proposed is presented. The main dynamic response of a membrane subject to sound pressure is introduced. In addition, a numerical study to evaluate the potentialities of this method is described. The main parameters influencing the tests are analyzed based on these numerical simulations. In the results and discussion sections, the methodology is applied to the analysis of a membrane similar to the tympanic membrane. Finally, the main conclusions are outlined.

## 2. Materials and Methods

The methodology of the experiment was developed to evaluate mechanical characteristics (Young’s modulus, pretension on membranes, damping, etc.) through the experimental and analytical correlation with a model test.

The configuration of the experiment must fulfill a series of requirements:

- The specimen has no added material.
- The test is non-destructive and repeatable with the same specimen.
- The specimen is subjected to a sound pressure that causes a vibration under the regime of small deformations.
- The configuration of the elements (source of sound, capture device, specimen, etc.) and the post-processing strategy must enable the detection of enough resonant frequencies to apply correlations.
- The test must reproduce a dynamic behavior in the specimen with a contrasted analytical solution.
- The experiment will be easy to reproduce with media currently available in standard laboratories.

### 2.1. Experiment Configuration

**Figure 1:**
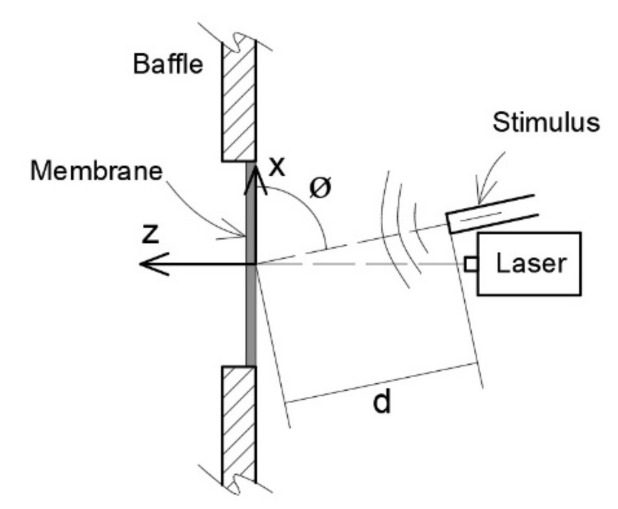
Configuration setup.

On Figure 1 we can see a schematic description of the experimental test proposed. The simplest configuration of the experiment was chosen: a sound source at 90°, and measurement of the center of the membrane. The sound source was placed at a distance d perpendicular to, and aligned with, the center of one of the faces of the membrane (angle of 90°). The harmonic experiment applies a stimulus through pressure to the membrane. The membrane specimen was placed into a baffle device with a free circular surface.

With this configuration, the axial symmetry vibration modes (1, 4, 9, etc.) can be obtained experimentally. With the data of the vibration modes, the Young’s modulus of the material can be calculated, which is the objective of the experiment.

### 2.2. The Shape of the Specimen. Analytical Correlation

The selected shape for specimens was circular. This is a common shape which is easy to reproduce with a device that serves as a support and leaves the circular portion of the specimen free.

**Figure 2:**
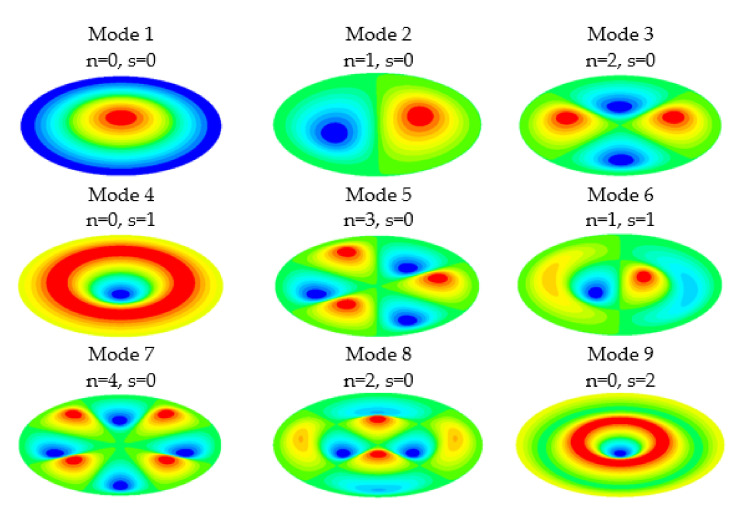
Modal shapes of the first 9 vibration modes.

There is also a theoretical, well-known relation between the frequencies of resonance and the mechanical properties of the material (Young’s modulus, Poisson’s ratio, density and geometry) for a circular membrane with clamped boundary conditions and no applied load. The equation to calculate the natural frequencies is [33]:

(1) $$f=\frac{\alpha }{2\pi a^{2}}\sqrt{\frac{\mathrm{gD}}{\gamma h}}=\frac{\alpha }{2\pi a^{2}}\sqrt{\frac{{Eh}^{2}}{12(1-\upsilon^{2})\rho }}$$

where *f* is the natural frequency in Hz, *a* is the radio of the membrane, *γh*/*g* is the mass per unit area, *D* is the flexural rigidity, *E* is the Young’s modulus, *h* is the membrane thickness, *υ* is the Poisson’s ratio, *ρ* is the density and *α* is a constant that depends on the number of nodal diameters *n* and the number of nodal circles *s* of the mode considered. Figure 2 reproduce the nodal diameters (*n*) and nodal circles (*s*) for the first nine modes of vibration of a circular membrane.

To identify frequencies, the experiment measured a selected number of points of displacement using a Laser Doppler Vibrometer (LDV, Polytec), then calculated the harmonic response and post-processed that information with an automatic Matlab software routine.

The different parameters that influences the resonant frequencies of the membrane, such as the dynamic response of an excitation, were studied. These parameters included the geometry of the membrane, damping and the position and arrangement of the excitation source, among others.

### 2.3. Measurement Objetive Region and Position of the Source. Numerical Analysis

The deformed shape of the membrane was different in each vibration mode. Figure 2 shows the shape of the first nine modes of vibration of a circular membrane. There were points of the membrane with large displacements compared to others. This issue must be taken into account in the choice of measurement points. The choice of which points to measure determines the modes that can be detected. In the experiment it was required to be able to conveniently excite the vibration modes we wanted to detect.

To study the influence of the parameters on the vibration modes, an extensive numerical study was carried out with the finite element software ANSYS. This methodology allowed us to determine the most suitable measurement points in the test, their position and the angle of the sound source.

Simulations of the harmonic response of a membrane with a diameter of 1 cm and different thicknesses were carried out. The mechanical properties of the membrane were: density 1200 kg/m^3^, Young’s modulus 2 GPa and Poisson’s ratio 0.35. Two positions of the source were considered: 90° and 45° between the axis of the source and the plane membrane. The distance from the sound source was 5 cm for a membrane of 40 µm thickness and 1 cm from the center of a membrane of 60 µm thickness. The harmonic response was compared with the modal response to analyse the influence of the combined parameters.

**Figure 3:**
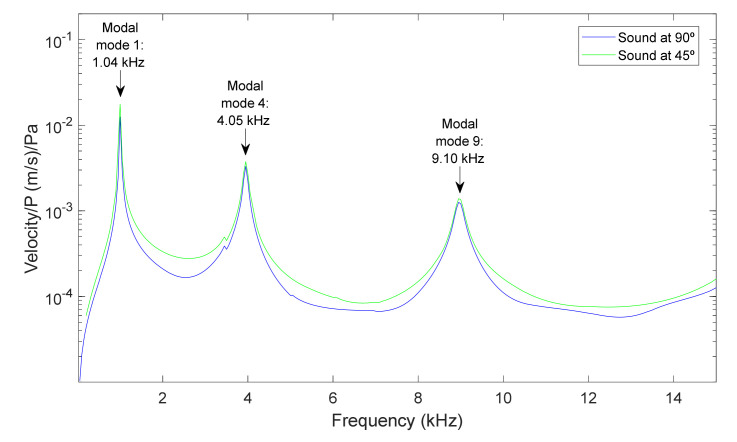
Center point of a membrane of 40 µm. Velocity magnitude at different positions of the source: (a) 90° and (b) 45°.

**Figure 4:**
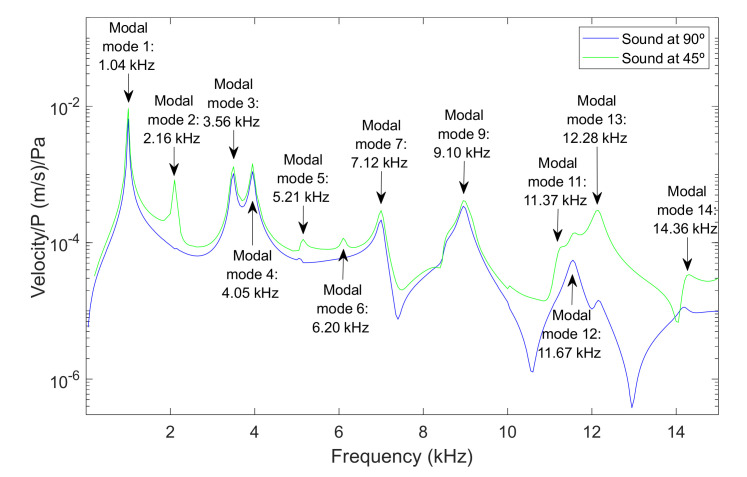
Point y = 0 x = radio/2 for a membrane of 40 µm. Velocity magnitude at different positions of the source: (a) 90° and (b) 45°.

Figure 3 and Figure 4 present the results for the membrane of 40 µm thickness, measured from two points of the membrane, at the center of the diameter and the point of coordinates y = 0 and x = radio/2, for a frequency range between 0.5 and 15 kHz, and for sound sources at 90° and 45°. The excitation that caused the response of the membrane was a sound wave originating from the source with a sound pressure of 1 Pa.

For the center of the membrane and the two positions of the source, it was observed that only the axial symmetry modes appeared, namely modes 1, 4 and 9. At this point, there was no significant influence from the position of the source. In the case of the point at y = 0 and x = radio/2, more modes of vibration were present in the response of the membrane. In the case of the position of the source at 45° almost all modal modes were observed. The selection of the position of the source in this controlled geometry allow us to involve more or fewer modes, depending on the data we needed to correlate properties of each specimen.

**Figure 5:**
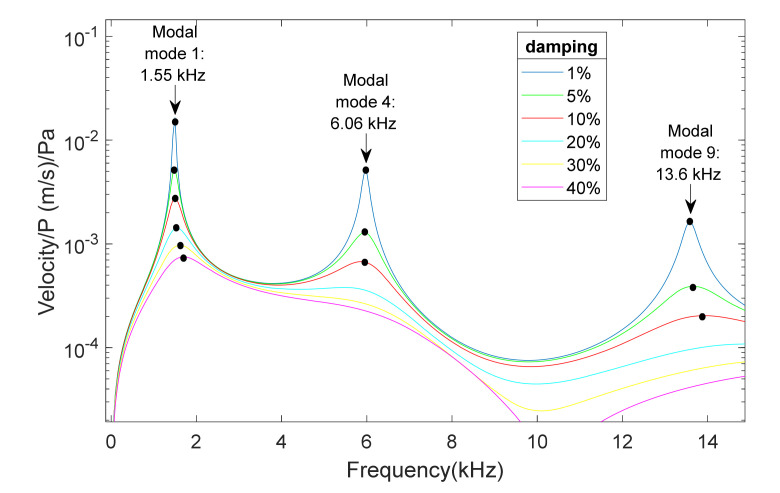
Center point for membranes of 60 µm. Velocity magnitude for the positions of the source at 90° for different damping values: 1%, 5%, 10%, 20%, 30% and 40%.

The influence of damping was also studied. Figure 5 shows data from one of the cases analyzed: the response of the membranes with thicknesses of 60 µm and an angle of the sound source of 90° from the central point of the membrane and damping values of 1%, 5%, 10%, 20%, 30% and 40%. Only the modes with axial symmetry were observed, as in Figure 3.

It was observed that, for larger values of damping, the peaks become softer, as expected. However, the peak values, which correspond to the resonant frequency for each case, generally moved to the right, increasing their values so the resonant frequency increases with increasing damping. For a high enough damping value, it is not possible to determinate the location of the peak of the graph. These simulations helped us to anticipate the number of potential main frequencies available for each type of specimen. In Figure 5, we detected three modes under 20% of damping. In higher-damping situations, we would recourse to reconfigure the test (position of the source, pressure level, etc).

**Figure 6:**
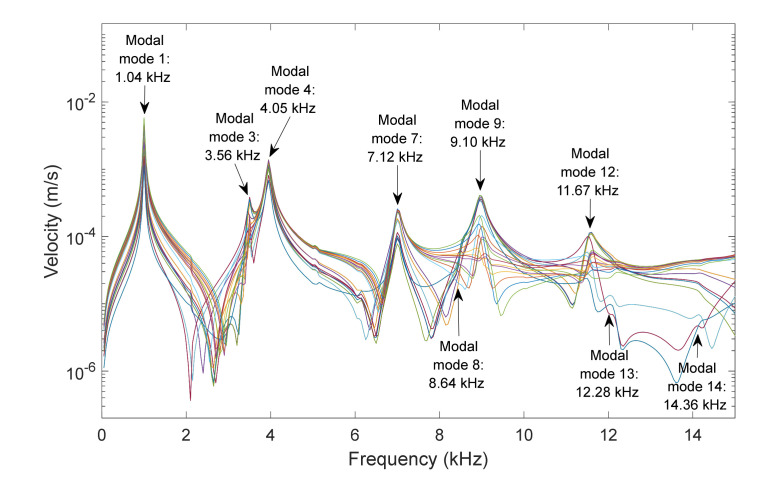
Surface responses of some representative points for a membrane of 40 µm, 90° source angle and 1 Pa of pressure in the source.

**Figure 7:**
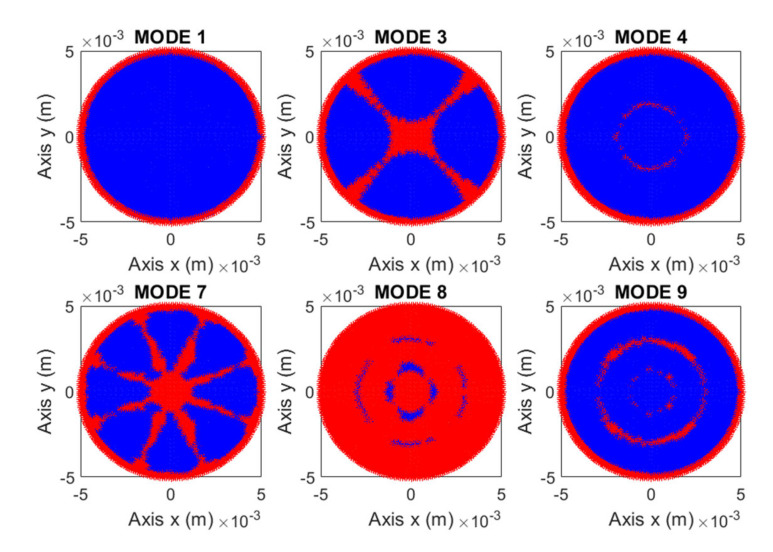
Candidate points from the surface of the membrane for a membrane with a thickness of 40 microns and with an angle of 90°. Modes 1, 3, 4, 7, 8 and 9.

Another important aspect to determine was the feasibility to detect the movement of the selected points. Figure 6 presents numerical results from some points of the surface of a membrane of 40 µm, with 90° source angle at 1 Pa. Velocity was selected as variable to evaluate the sensibility needed from the Laser Doppler Vibrometer, depending on the point analyzed. A value of 2·10^−5^ m/s was established as the minimum velocity for a good detection of movement by a standard LDV. Figure 7 presents the points of the surface that met the condition of being accurately detectable by LDV in the simulation configurations for the first six modes that could be observed with these configurations: modes 1, 3, 4, 7, 8 and 9. This routine allowed us to adjust the combined pressure level and LDV sensibility in each experiment.

### 2.4. Circle Fit Method

The correlation among the natural frequencies and the FRF was based on the visual observation of the maximum peaks of the response. This was an intuitive procedure, which is valid when numerical data were used with a low level damping, especially for illustrative purposes. Nevertheless, experimental data showed higher damping and complexity, and we resorted to numerical curve-fitting techniques to identify the resonance frequencies.

In this particular case, we used the classical circular fit method, which is used for the calculation of vibration modes and damping [31] and is based on the use of the Nyquist diagram. Additional FRF curve-fitting techniques were explored, but this simple method was proven to be valid for the purpose of this work.

In any case, it must be stated that the use of the classic FRF curve-fitting techniques implies the admission of certain assumptions. These techniques are used in the field of experimental modal analysis and their validity in this case is not strictly justified. They are based on the curve-fitting of FRF, which is considered as the mobility response (normally in terms of acceleration) to a force applied. Both parameters were required to be measured in the experiment. 

In this specific problem, the output was easily measured but the input could not be obtained because it was a spatial pressure distribution along the membrane. The parameter that we could measure in this experiment was the sound pressure at a certain point close to the sound source. In this paper we refer to FRF as the ratio of the mobility output to the sound pressure (instead of mobility output and force applied). As we will see, this assumption was acceptable.

The circular fit method was based on the Nyquist diagram where the presence of a natural mode of vibration can be seen because the function takes the form of a circumference. Its size and position depend on the different parameters that characterize the mode (natural frequency, damping coefficient, etc.), as well as on the rest of the existing modes in that function.

The method consists of fitting a circle to a complex plane using the least squares model. For the method to be valid, several requirements must be met as that the damping is small and that the modes are decoupled.

Equation (2) represents the displacement frequency response function, where *ν_k_* is the natural frequency, *αk* is related to the damping coefficient, *U_k_* + *iV_k_* is the residual of this mode, and *R_k_* + *iI_k_* represents the influence of the rest of the modes.

(2) $$H(\omega )=\frac{U_{k}+iV_{k}}{i\omega -\alpha_{k}-i\nu_{k}}+R_{k}+iI_{k}$$

Damping can be calculated with Equation (3) [36]. In this equation, *ρ* is the angle formed by a point on the circumference (with a frequency *w*), with the point of the modal frequency (*ν_k_*), provided that the angle is not greater than 90°.

(3) $$\alpha_{k}=\frac{\omega -\nu_{k}}{tg\frac{\rho }{2}}$$

**Figure 8:**
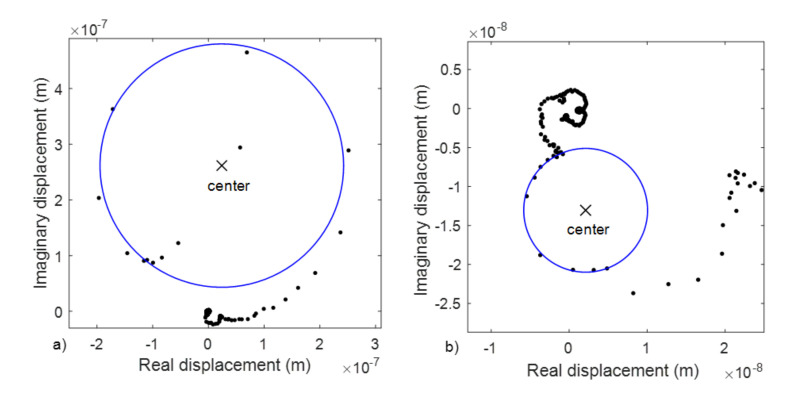
Nyquist diagrams. Application of the circular fit method for two resonance frequencies (**a**,**b**).

In Figure 8 the Nyquist diagrams for the points obtained in the experimental test with two examples of circular fit are presented.

## 3. Experimental Results

The method used to correlate mechanical properties and frequency parameter based on numerical data was straightforward. Nevertheless, its ability remains to be proven based on real experimental data. In experimental tests, many aspects come into play, especially when the manipulation of small samples is necessary, the magnitude to measure is small and the setup presents many parameters to consider. The main goal of the present work was to check the validity in practice of the assumptions made.

### 3.1. Experiment Setup

**Figure 9:**
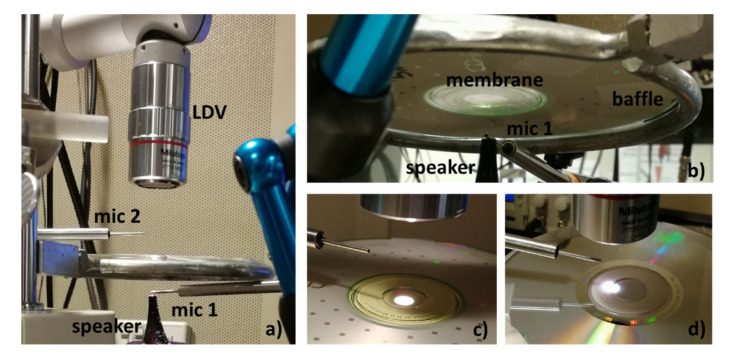
Disposition of the different elements in the experiment (**a**,**b**). Measurement at the center of the membrane (**c**). Measurement at a support point (**d**).

According to the experiment setup described in the previous section, several experiments were implemented. They were performed in the VA Loma Linda Healthcare System, CA, USA. Figure 9 shows the setup of these experiments. The membrane was supported by a structure that acted as a baffle. The membrane was in a horizontal position and the speaker was placed below the membrane at a distance of 1 cm. The velocity was measured using an LDV placed on the opposite side. The figure shows both spots used in this paper: the center of the membrane and the external border. Two Sokolich ultrasound microphones were used, one to calibrate the sound pressure at the speaker and to monitor the sound pressure level at the speaker output (mic 1) and another to capture sound transmission (mic 2). The first microphone was used as the input signal while the second was not used in this present work.

Sound-induced membrane velocities were measured from two perpendicular lines across the center of the membrane with many locations along the line and a location resolution of 0.2 mm, using a Laser-Doppler Vibrometer (LDV, OFV-5000, Polytec Inc., Germany). Stimulus and acquisition programs were written in Matlab and TDT Visual Design Studio. The sampling frequency of the TDT system was 200 kHz. Pure tones (1–2 s duration) were generated by a Tucker Davis Technology (TDT) system III. Data were stored following the removal of the first 4,096 points of the response waveform to avoid the transient, and after time-averaging the remaining waveform, typically in 50 time-locked segments. The responses were later analyzed by Fourier transform in Matlab. The frequencies were swept from 0.5 to 15 kHz in 500 Hz steps.

The material used was parafilm. It is a membrane material commonly used in laboratories to seal recipients. Its thickness and mechanical properties make it ideal for this experiment due to some similarity to the tympanic membrane. According to the manufacturer information the membrane thickness was approximately 130 microns, and its density was 992 kg/m^3^. No information was provided about is elastic modulus.

Three different samples were prepared with circular membranes with diameters of 5, 10 and 15 mm. The membranes were clamped between two discs with a hole corresponding to the desired diameter. The discs acted as baffles, such that the sound pressure only pushed one of the membrane sides.

The velocity of the central point of the membrane was measured. The support system was also measured as control. Sound pressure was applied at an approximate distance of 1 cm, with an angle Φ of 90°. This corresponded to the simplest setup possible, leading to a theoretical axial symmetric response. The excitation frequencies ranged from 0 and 10 kHz for the 10 and 15 mm diameter membranes, and from 0 and 15 kHz for the 5 mm membrane due to its higher natural frequencies. The frequency sample was Δf = 47.7 Hz for all the cases.

### 3.2. Experiment Results

**Figure 10:**
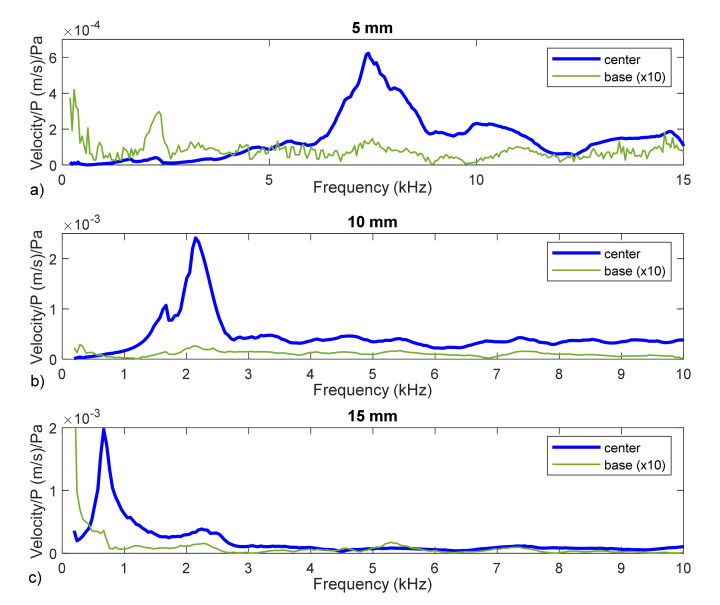
Membrane velocity transfer function. Responses of membranes with diameters of 5 mm (**a**), 10 mm (**b**), and 15 mm (**c**) for the center location (blue curve) and edge location (green curve). Responses at the edge location are scaled up to 10 x.

The results obtained for the different cases are plotted in Figure 10. They corresponds to the transfer function of the velocity at the membrane divided by the speaker sound pressure (input, mic 1). Results corresponding to the support system (border) were also plotted and it will be of great interest to interpret the results as it will be explained later. It was magnified (×10) to facilitate comparison.

Visually, we could identify some maximum that we could interpret as being linked to the natural frequencies. For each diameter, a clear first resonance was observed (at 7360, 2124 and 667 Hz, respectively). Nevertheless, it was difficult to distinguish some additional maximum at higher frequencies. So, we resorted to evaluating these frequencies by means of curve fit methods, particularly by means of the circular fit method. Additionally, as will be shown, the visually detected frequencies were proven to be erroneous.

### 3.3. Circular Fit Results

As described in previous section, the circular fit method is based on the Nyquist diagram, where the presence of a natural mode of vibration can be identified as a circumference on the FRF. This characteristic is correct in the case of a mechanical system where the FRF is defined as displacement divided by force applied. In our case, we cannot measure the force applied, we only have the sound pressure at a certain distance (mic 1). Thus, the use of this methodology could be questionable. As we saw in this experiment, this limitation can be overcome, and we obtained resonant frequencies with great accuracy.

**Figure 11:**
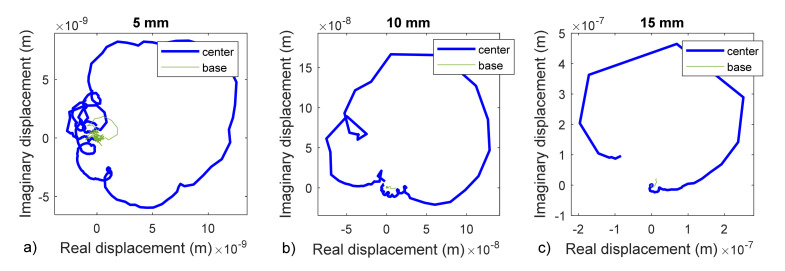
Nyquist diagram for membranes with diameters of 5 mm (**a**), 10 mm (**b**), and 15 mm (**c**) for the center location (blue curve) and edge location (green curve).

In Figure 11, the results shown in Figure 10 are plotted in terms of displacements in the Nyquist diagram. Circular shapes can be clearly observed in all the cases, even for the base data.

A semi-automatic sweep procedure was applied to identify circular fits for the data shown in Figure 11. Table 1 and Table 2 show the most significant values obtained for each case, at the center of the membrane and at the border, respectively.

**Figure 12:**
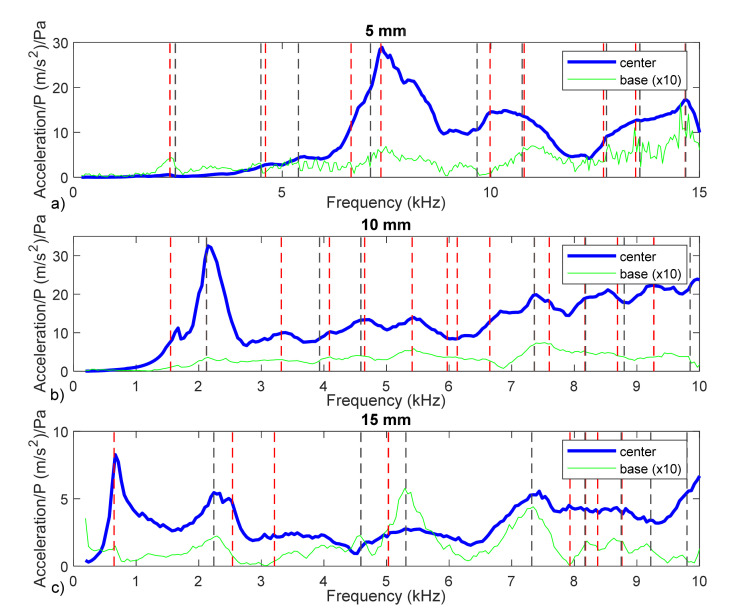
Membrane acceleration transfer function. Responses of membranes with diameters of 5 mm (**a**), 10 mm (**b**), and 15 mm (**c**) for the center location (blue curve) and edge location (green curve). Resonant frequencies added at the center (red line) and at the border (grey line).

Many frequencies were detected on the border of the support device. Some of these frequencies were present at the center of the membrane and some were not. In Figure 12, the results obtained are plotted in terms or acceleration. This magnitude provides higher values at higher frequencies, allowing us to observe the phenomenon present at a higher range of frequencies. In this figure, vertical lines were added at the frequencies calculated by circular fit method, for the center of the membrane (red) and for the border (grey).

## 4. Discussion

Analyzing the data plotted in Figure 12, we determined the capabilities and limitation of the methodology proposed. Among others, we can highlight the following issues:

- Many frequencies were detected, more than the number of expected frequencies, considering the numerical model of the problem (Figure 2).
- Some of the frequencies were coincident approximately with the observed peak, both at the membrane and at the border.
- Some other frequencies appeared where no clear observable maximum was present.
- When the frequencies were close to peaks, they were not the same values.

To understand all these aspects, we evaluated each of them in order to extract the data useful for our purpose.

The first point to comment on is the presence of resonant frequencies on the support of the membrane. The design of the experiment did not expect significative values at this location. Actually, the magnitude measured was very low (on the figures it was magnified ten times). Some peaks can be seen but it was expected that their influence on the vibration of the membrane could be negligible. Nevertheless, one finding was that they have influence on the response of the membrane.

In Figure 12, it can be observed that the same frequencies (or very close frequencies) are present in the response of the membrane and the border (close magenta and cyan lines). This means that the membrane vibrates in response to a mechanical input (the displacement of the base) instead of the acoustical input.

It can be seen that a group of frequencies was present in the three experiments with slightly similar frequencies: 2200, 4500, 7300 and 9750 Hz (see Table 2). This was probably due to the structural dynamics behavior of the whole experimental system being related to natural frequencies of this system. This effect is not desirable, and future experiments should be designed to eliminate it.

In any case, it is not a real limitation, because we can ignore these frequencies and consider as valid natural frequencies of the membrane (the goal of the experiment) only the remainder of the frequencies shown in Table 1.

Thus, the first frequency for each case would be 6652 Hz (5 mm), 1552 Hz (10 mm) and 648 Hz (15 mm). These frequencies are not exactly the maximum peaks observed in Figure 10 or Figure 12. In the 15 mm case, the difference was small (648 compared to 667 Hz) and was due to the influence of damping, as stated in the previous section. In the other cases, the resonant frequencies of the structural system are masking the membrane’s natural frequencies and can lead to a wrong interpretation of the data. As a rule, for future tests, the measurement of the supporting structure should be required to obtain valid results.

The ability of the circular fit method to detect properly these values and many others impossible to detect visually was remarkable. More complex curve fitting methods that we are currently evaluating exist, but the results provided with this simple method are very satisfactory.

Now that we have the natural frequencies for each diameter (those marked with an asterisk in Table 1), we can use them to evaluate the mechanical properties of the material. In this experiment, we have an acceptably accurate knowledge of the main parameters of the material and the membrane (density, diameter and thickness). The only parameter left to identify is the Young’s modulus (E). As the material is the same, the value calculated must be the same for the three diameters.

**Figure 13:**
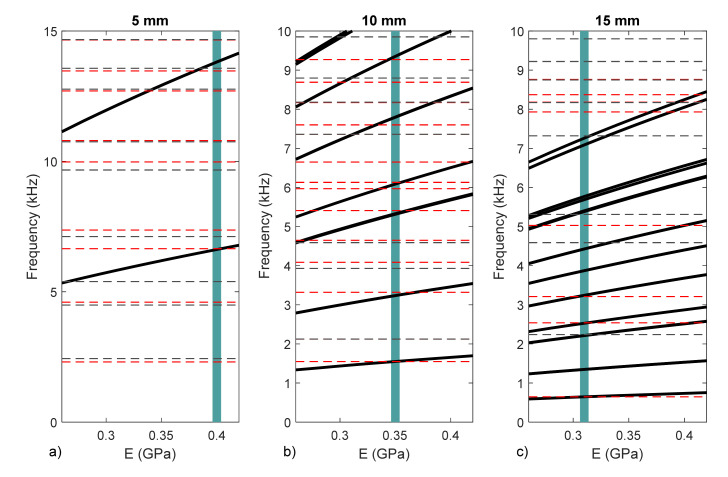
Young’s modulus determination for membranes with diameters of 5 mm (**a**), 10 mm (**b**), and 15 mm (**c**).

For this purpose, we calculated the natural frequencies for membranes with those diameters, but changing the value of the Young’s modulus (in a range from 2.6 to 4.4 GPa). The results obtained are shown in Figure 13. It can be seen how the natural frequencies increase with E (black lines).

Then, we can plot the frequencies obtained experimentally as horizontal lines. Crossing these lines with the numerical results, we can obtain the value of E coherent with the experiment results.

In the first case, 5 mm, we see that we only have one measurement by which to identify the value of E. The first natural frequency provided a value of E = 0.4 GPa. A second crossing point with the second natural frequency at 13.4 KHz appeared, but we could not consider it valid as it also appeared on the base movement. In this case, we can estimate a value for E, but we do not have a second point to validate. The high stiffness of this small membrane provides very high frequencies, difficult to measure from a practical point of view.

For the 10 mm case, we have more frequencies in the range of the experiment. Therefore, we have more valid crossing point. The experimental results cut f_1_, f_2_, f_3_, f_4_, f_5_ and f_6_. The exact point was slightly different for each frequency, but we can consider that the value E = 0.35 GPa produces less than a 5% error. Due to the axial symmetry configuration of the experiment, we only expected to obtain f_1_ and f_4_. As this condition is difficult to reproduce in practice, we obtained more information than expected. In summary, this proved to be the best experimental setup to obtain the value of E.

Finally, in the 15 mm case, we had valid cuts with f_1_, f_4_ and f_5_, and a value of E = 0.31 GPa was obtained. In this case, fewer frequencies were detected in the low frequency range, probably the experiment was centered more properly, and we had no information from f_2_ and f_3_. Many higher frequencies were detected that were difficult to distinguish from the base frequencies and correlate with the higher modes.

The variability on the results obtained was interesting. Young’s modulus was obtained in a broad range (0.31−0.4 GPa). One of the reasons for this variability is that probably there is a certain heterogeneity of the material (parafilm) and different samples can lead to different responses. If necessary, this aspect can be clarified in future with additional tests.

Nevertheless, the more interesting at this point is to analyze where these discrepancies are coming from and how can we limit them in future experiments.

Not all the experiments had the same validity. The results obtained with the 10 mm membrane proved to be the best suited for this test. 

In the case of the 5 mm membrane, we only had one measurement by which to estimate E; if there was some small deviation, we could not detect it. The range of frequencies of the experiment was short. Additionally, we must consider the influence of small uncertainties in the known parameters, such as geometry, density, etc. We will focus on the diameter parameters. If we consider the numerical model to compare, there was another fact that could have altered the result. In the numerical model, we can introduce an exact diameter and ideal boundary conditions (fixed ends). Both conditions are intimately linked, and were difficult to distinguish in the real experiment, especially for smaller sizes. With a smaller diameter, small errors on the diameter size estimation leads to a broad range of E uncertainty. Suppose a 0.5 mm error in the three cases under study, the range of uncertainty would be a 10% in the 5 mm case and 3% in the 15 mm case, and the natural frequencies are inversely proportional to the quadratic value of this parameter (Equation (1)). A total of 0.5 mm of uncertainty in this experiment is a credible estimate, considering the practical difficulties of affixing the sample on the support.

Another aspect to consider is the frequency sample of the data. It was the same for all the cases (47.7 Hz). If we calculate the ratio f_1_/Δf we would have an idea of the discretization level of the FFT, which would be the worst in the case of the 15 mm membrane. This can be observed in the Nyquist diagram in Figure 11, where the circles are more easily identified with the lower diameter membranes than with the 15 mm membrane.

Based on this discussion and the experiment results, we concluded that a range of values from 0.31 to 0.35 GPa was more reliable in this case, probably closer to 0.35 GPa.

## 5. Conclusions

A new methodology to obtain the mechanical properties of a membrane was shown. The numerical bases were stated and a particular experiment was used to check the validity of the procedure.

The three different configurations of the experiment provided information to prove the utility of the method and to evaluate key points for improvement.

The Young’s modulus was identified for the material tested with three different setups. A certain variability was observed in the results, but the general procedure was considered valid. The difficulties shown during the experimental tests have revealed key aspects that must be considered in the design of future experiments.

Among others, the influence of the sample size on the uncertainty of the procedure or the vibration of the support system that contaminated the output signal were highlighted and explained.

It was proven that the use of classic FRF curve-fitting techniques is applicable to this experiment even when the input measured is the sound pressure from a certain distance instead of the force applied to a structure. The circular method proved its utility. Other FRF curve-fitting techniques are being investigated to improve the results.

## Figures and Tables

**Table 1:** Resonant frequencies detected by circular fit method at the center of the membrane (Hz).

5 mm	10 mm	15 mm
2312	1552	648.5
4602	2124	2545
6652	3322	3210
7366	4087	5031
9985	4649	7934
10848	5415	8179
12709	5973	8373
13472	6132	8757
14662	6649	
	7366	
	7612	
	8173	
	8685	
	9272	

**Table 2:** Resonant frequencies detected by circular fit method at the border (base) of the membrane (Hz).

5 mm	10 mm	15 mm
2439	2124	2240
4492	3933	4594
5396	4599	5315
7116	7366	7320
9669	8179	8173
10751	8798	8752
12771	9853	9225
13572		9798
14677		

## Data Availability

The data presented in this study are available on request from the corresponding author. The data are not publicly available due to needs the permission of principal investigators of each research project involved.
